# Challenges in the quality assurance of elemental and isotopic analyses in the nuclear domain benefitting from high resolution ICP-OES and sector field ICP-MS

**DOI:** 10.1007/s10967-015-3952-5

**Published:** 2015-02-14

**Authors:** Michael Krachler, Rafael Alvarez-Sarandes, Stefaan Van Winckel

**Affiliations:** European Commission - Joint Research Centre, Institute for Transuranium Elements, ITU, P.O. Box 2340, 76125 Karlsruhe, Germany

**Keywords:** Quality assurance, ICP-OES, ICP-MS, Nuclear fuel, Reference materials

## Abstract

Accurate analytical data reinforces fundamentally the meaningfulness of nuclear fuel performance assessments and nuclear waste characterization. Regularly lacking matrix-matched certified reference materials, quality assurance of elemental and isotopic analysis of nuclear materials remains a challenging endeavour. In this context, this review highlights various dedicated experimental approaches envisaged at the European Commission—Joint Research Centre—Institute for Transuranium Elements to overcome this limitation, mainly focussing on the use of high resolution-inductively coupled plasma-optical emission spectrometry (HR-ICP-OES) and sector field-inductively coupled plasma-mass spectrometry (SF-ICP-MS). However, also α- and γ-spectrometry are included here to help characterise extensively the investigated actinide solutions for their actual concentration, potential impurities and isotopic purity.

## Introduction

Over the last decades, analytical quality assurance has gained in importance in many scientific areas, including the analysis of radioactive specimens. The use of certified reference materials is an important pillar for the assessment of the quality of any acquired analytical data. Such matrix-matched certified reference materials that are employed frequently for both quality control and method validation are unfortunately not available for most investigations relevant to the nuclear domain [1]. Therefore, it would be most helpful to compare the analytical results obtained for a particular instrumental technique, e.g. inductively coupled plasma-mass spectrometry (ICP-MS), with data from another methodology whose analyte detection is based on a different physical principle, e.g. inductively coupled plasma-optical emission spectrometry (ICP-OES). Both before mentioned techniques work independently because the separation/detection of elements or more specific isotopes is based on the mass-to-charge ratio (m/z) in ICP-MS, while emission of light of element characteristic wavelengths is fundamental to ICP-OES.

Using such complementary experimental approaches reduce largely the likelihood of the occurrence of identical analytical problems related to the determination of a specific analyte leading to either a positive or negative bias of the final result. Two particular examples may highlight this important aspect: First, the presence of a large excess of ^238^U in the analyte solution always hampers the reliable determination of ^237^Np (being a direct neighbour of ^238^U in the mass spectrum) using ICP-MS. Depending on the employed ICP-MS instrument, the abundance sensitivity, i.e. the impact of the peak tailing of ^238^U on the ^237^Np signal, is slightly different, but leads to a positive bias of the acquired Np data at all times. For ICP-OES, in turn, suitable emission wavelengths have been identified that allow an accurate determination of ^237^Np in the presence of ^238^U [2]. Second, isobaric interferences occurring in ICP-MS, e.g. ^238^Pu and ^238^U or ^241^Am and ^241^Pu, cannot be resolved spectroscopically, even using sector field (SF-)ICP-MS [3]. Again, these analytical problems are overcome by using appropriate ICP-OES emission wavelengths for the particular analytes of interest because the above mentioned isotopes emit light at different wavelengths [3, 4].

Moreover, this cross-validation of two independent analytical procedures helps to identify potential limitations of a specific analytical method envisaged to analyse a selected element/isotope. An agreement of results obtained by at least two independent analytical methods, in turn, essentially improves the creditability of the acquired analytical data. In addition, such inter-method comparisons are carried out preferably in-house for radioactive samples basically because of the huge efforts necessary with respect to safety, security, and monetary aspects associated with a transport of such specimens to another external laboratory.

Whenever possible, the analysis of nuclear samples may be carried out directly, i.e. without the need of chemically separating off the analyte of interest from the remaining elements [2–5]. This straightforward approach is not only faster, but also results in less radiation dose originating from the sample to the laboratory personnel. The presence of a variety of fission products and minor actinides in the analyte solution, however, complicates frequently the application of a particular instrumental approach. Consequently, complementary analysis employing diverse instrumental techniques based on different physical detection principles is a key issue to ascertain the reliability of the generated analytical data, especially when “non-separated” fuel solutions, containing all fission products as well as actinides, are to be analysed.

Currently, the potential of high resolution (HR-)ICP-OES for elemental and isotopic analysis in the nuclear domain is not fully exploited, often leading to modest performance only [6–13]. Even though early work already demonstrated the successful application of this analytical technique to the nuclear field about 3 decades ago [6–9, 14], this knowledge disappeared in some way with the introduction of commercial ICP-MS instruments in the early 1990s. With the wide spread of ICP-MS, being more sensitive and providing superior performance in terms of isotopic analysis, investigations on the potential of ICP-OES for this kind of analysis largely stopped.

At the European Commission—Joint Research Centre—Institute for Transuranium Elements (EC-JRC-ITU), we aim at reconsidering this powerful analytical technique for both elemental and isotopic analysis of actinides and fission products in a substantial variety of samples within the nuclear domain. In addition, its potential to complement other well established analytical techniques such as ICP-MS, as well as α- and γ-spectrometry are highlighted in this study.

The main intention of this review is to summarize and share the experience gained during the in-house analysis of nuclear samples, thereby also raising the awareness of the importance of analytical quality assurance in the nuclear field. Using a commercial HR-ICP-OES spectrometer, sensitive emission wavelengths for potential isotopic and elemental analysis of nuclear samples were identified and inspected thoroughly. Besides, analytical procedures based on SF-ICP-MS were developed and applied subsequently to cross-validate the HR-ICP-OES results. The benefits and pitfalls of different quantification strategies applied to HR-ICP-OES and SF-ICP-MS analysis were examined carefully, complemented by α- and γ-spectrometry measurements. Specific innovative examples presented here include (1) U isotopic analysis with HR-ICP-OES, identifying depleted, natural and enriched (at various levels) abundances of ^235^U; (2) the accurate determination of alkaline elements, neodymium (Nd), and neptunium (Np) concentrations in nuclear specimens including samples from pyrochemical treatment of spent fuel; as well as (3) the direct elemental and isotopic analysis of americium (Am) in non-separated spent fuel solutions.

## Analytical performance of high resolution ICP-OES

In contrast to conventional ICP-OES instruments equipped with a charge coupled device (CCD) detector for fast, simultaneous analysis, the sequentially working HR-ICP-OES employed in our studies benefits from a photomultiplier. Among the main differences between conventional and HR-ICP-OES is the fact that the latter consists of a superior optical path allowing an advantageous separation of individual emission wavelengths from each other. While conventional ICP-OES instruments with a CCD typically provide optical resolutions in the range of 10–20 pm (depending on the wavelength region) [13, 15], this value is well below 5 pm for most emission wavelengths of interest for HR-ICP-OES [11, 16]. The superior high optical resolution of the latter allows for measurements of peak increments of <0.5 pm and better identification of potential spectral interferences.

Normally used in its standard configuration (pneumatic nebulizer, sample uptake rate of ~1–2 ml min^−1^), reported ICP-OES detection limits (LODs) for the determination of selected minor actinides and fission products are in the low to mid µg kg^−1^ range, depending on the element considered [6–11]. While these LODs are sufficient for a number of applications in the nuclear field [10–13], instrumental performance can be improved substantially providing some distinct benefits as described in more detail below.

**Fig. 1:**
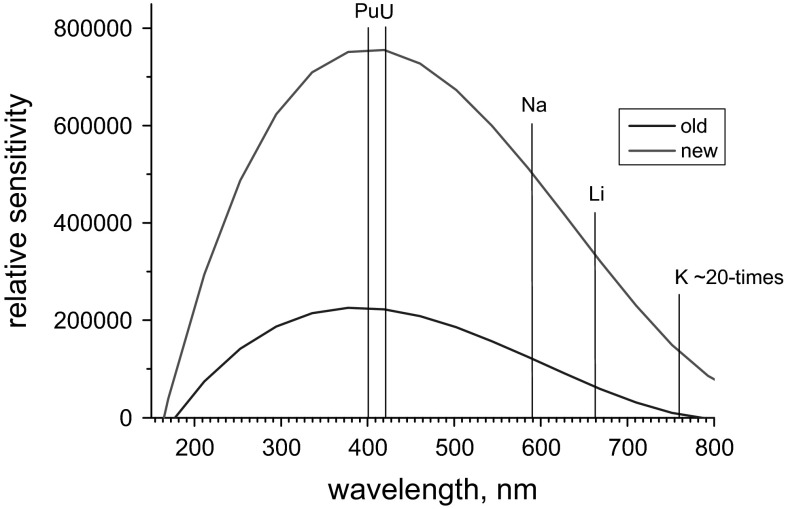
Comparison of the performance of two diverse detectors used with the identical HR-ICP-OES instrument. *Old* refers to the commonly employed standard photomultiplier, whereas the *new* detector denotes a high sensitivity multialkali photocathode providing high near-infrared sensitivity. In addition, typical wavelengths of selected elements measured frequently at EC-JRC-ITU employing ICP-OES are indicated

To push the detection power of the HR-ICP-OES instrument (Ultima2, HORIBA Jobin Yvon, Longjumeau, France) used in our studies, the standard photomultiplier detector (R446, Hamamatsu Photonics, Shimokanzo, Japan) was replaced by a commercial high sensitivity multialkali photocathode (R955, Hamamatsu Photonics) [17]. This substitution led to a minimum 3-fold enhancement of the instrumental response with up to 20-times improvement for elements assessed in the upper wavelength region, e.g. K at *λ* = 766.490 nm (Fig. 1). Additionally, a high efficiency desolvating nebulizer (Apex E, Elemental Scientific (ESI), Inc., Omaha, NE, USA) operated at a low sample uptake rate of ~0.3 ml min^−1^ was employed increasing substantially sample transport efficiency [17]. The above mentioned upgrade of the HR-ICP-OES spectrometer resulted in superior LODs that were approximately one order of magnitude lower compared to the original instrumental set-up across the entire wavelength range. This development allowed for higher dilution factors of the original sample solutions, resulting in less radiation dose to the operator. The applied approach also required less sample volume, and as such, produced less radioactive waste that is expensive to dispose of.

Using this advanced instrumental set-up, we showcase selected recent applications of HR-ICP-OES analysis of nuclear specimens carried out at EC-JRC-ITU and demonstrate its potential for cross-validating other well-established analytical techniques such as SF-ICP-MS (Element2, Thermo Scientific, Bremen, Germany).

## Nuclear forensics—uranium isotopic analysis

**Fig. 2:**
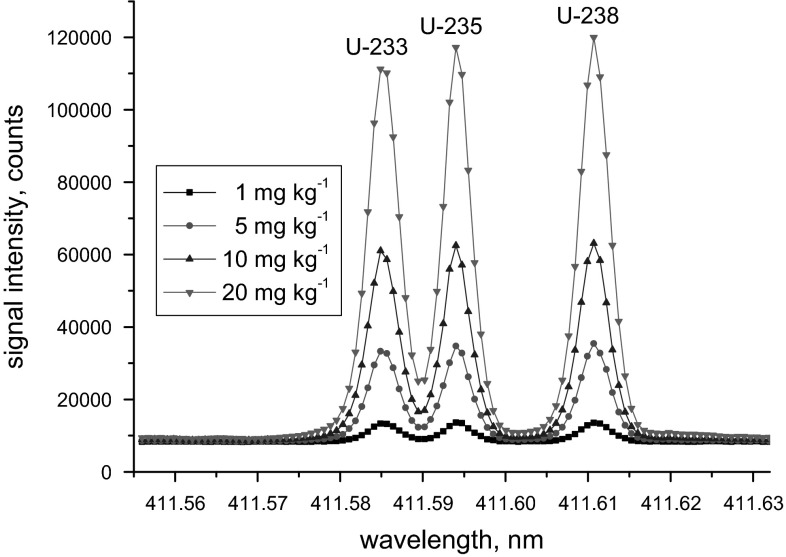
HR-ICP-OES spectra of the certified reference material EC-NRM-199 having almost identical isotope amount fractions of ^233^U, ^235^U, and ^238^U. Spectra have been recorded using the optimised instrumental set-up (see text for details) at four different concentration levels. Reported concentrations refer to the total amount of U, i.e. concentrations of individual U isotopes amount to only ~1/3 of this total U concentration

Against other opinion [18], ICP-OES analysis can also provide valuable isotopic information (Fig. 2). This feature is based on the so-called isotopic shift caused by atomic transitions of various isotopes of the same element emitting light at slightly different wavelengths. Corresponding pioneering work has been carried out as early as 1981 [14] being later on applied to the nuclear field [16, 19–24]. The large isotopic shift of 25 pm between the ^235^U and ^238^U ICP-OES emission signals at *λ* ~ 424.4 nm, for example, allows for the analysis of depleted, natural and enriched U samples [24]. Benefitting from the high optical resolution of HR-ICP-OES of <5 pm, baseline separated emission signals for the two U isotopes are achieved easily. As highlighted in Fig. 2, even the emission signals of ^233^U, ^235^U, and ^238^U can be separated from each other without difficulty at *λ* ~ 411.6 nm (but also at *λ* ~ 424.4 nm) using commercial HR-ICP-OES instruments [16].

The particular enrichment of U in a sample is determined via the abundance of ^235^U. To this end, net peak intensities of both ^235^U and ^238^U emission signals are considered. The accuracy of such ICP-OES analysis can be assessed straightforwardly by comparing the ICP-OES data with results obtained from mass spectrometric techniques such as multi collector (MC)-ICP-MS or thermal ionisation mass spectrometry (TIMS) (Table 1). The successful application of this analytical procedure to the field of nuclear forensics, helped identifying enriched U (up to 90 %) in a shipment of scrap metal from outside the EU entering an European harbour some time ago (Table 1). Employing γ-spectrometry, routine on-site screening revealed already elevated levels of radiation in this scrap metal. Sample analysis using HR-ICP-OES at EC-JRC-ITU confirmed largely the preliminary on-site screening results, however, with much higher precision. More important, comparative in-house MC-ICP-MS and TIMS analyses confirmed the accuracy of the HR-ICP-OES results.

**Table 1:** Uranium isotopic analysis ^235^U enrichments [%] of various scrap metal samples as assessed using various instrumental techniques

Sample	γ-spectrometry	HR-ICP-OES	MC-ICP-MS^a^	TIMS^b^
A	7.8 ± 0.5	9.2 ± 0.1	9.0380 ± 0.0079	9.0333 ± 0.0052
B	47.6 ± 5.9	45.6 ± 0.2	45.838 ± 0.033	45.847 ± 0.017
C	42.4 ± 2.1	43.7 ± 0.2	43.811 ± 0.063	43.800 ± 0.016
D	90.2 ± 4.2	90.0 ± 0.1	89.689 ± 0.017	89.730 ± 0.036

^a^Multi collector-inductively coupled plasma-mass spectrometry

^b^Thermal ionisation mass spectrometry

Among the advantages of using HR-ICP-OES for the reliable assessment of ^235^U enrichments is the fact that no matrix separation is required prior to analysis. As such, HR-ICP-OES can be employed as a fast screening tool, providing sufficiently accurate (<1.5 %) and precise (~1 %) U isotopic information [24]. Setting up necessary laborious chemical separation procedures for subsequent mass spectrometric measurements can benefit from such overview HR-ICP-OES analysis. Compared to most mass spectrometric techniques, no so-called “mass bias” correction is required for HR-ICP-OES analysis of U isotopes. In addition, the main assembly of the HR-ICP-OES does not get contaminated radioactively, a crucial issue that cannot be avoided using mass spectrometry. Because the difference in signal intensity of the minor (^235^U) and major abundant (^238^U) U isotopes is only about two orders of magnitude at most, no deteriorating effects limiting the intensity linearity are observed [24]. If U isotope ratios would become larger, then worsening effects such as self-absorption might hamper the reliable determination of U isotopes using HR-ICP-OES. This kind of measurement, however, is beyond the instrumental capabilities of HR-ICP-OES [24].

In addition to comparative measurements, certified reference materials such as the IRMM 184–187 series (Joint Research Centre, IRMM, Geel, Belgium) or NBS CRM U100, U500, and U850 (New Brunswick Laboratory, Argonne, IL, USA), are available for quality assurance [24]. However, it is important to note that for many nuclear applications matrix-matched certified nuclear reference materials (e.g. spent fuel) are not available [1].

## Pyrochemical treatment of spent nuclear fuel

Among several separation concepts being developed worldwide, pyrochemical treatment of spent fuel is an experimental approach aiming at minimizing the amount and radiotoxicity of both spent nuclear fuel and radioactive waste [25]. Most pyrochemical separation processes are based on electro-refining of metallic fuel dissolved in molten salts, e.g. LiCl–KCl eutectic mixtures [26]. For process optimisation, both salt mixtures and elements/isotopes deposited on the electrodes need to be analysed. Certified matrix-matched reference materials are currently not available for quality control of such analysis.

In that context, the analysis of ^237^Np, a representative of the minor actinides, is frequently required. Many analytical procedures—including ICP-OES—depend on the availability of a well characterised ^237^Np stock standard solution in order to calibrate the instrumental response [2]. Once a ^237^Np solution is at hand to become a calibration standard, its accurate ^237^Np concentration needs to be assessed reliably. This characterisation can be readily achieved using γ-spectrometry employing selected γ-ray emission energies from both ^237^Np (direct measurement) and its daughter ^233^Pa (indirect measurement). Once ^237^Np and ^233^Pa are in secular equilibrium, both measurement strategies must result in a similar Np concentration [2]. Therefore, this experimental approach already serves as an internal quality control check, the outcome of which can be confirmed additionally by using another independent method such as SF-ICP-MS (Table 2).

**Table 2:** Cross-validation of γ-spectrometry and SF-ICP-MS measurements for the assessment of the concentration of a ^237^Np stock standard solution [2]

Analytical technique	^237^Np, mg kg^−1^
γ-spectrometry *directly* via ^237^Np	1,209 ± 23
γ-spectrometry *indirectly* via ^233^Pa	1,236 ± 7
SF-ICP-MS	1,233 ± 10

As there is only a few literature data available [8, 9, 27], such a well characterised ^237^Np standard solution can be employed to identify the corresponding most sensitive ICP-OES emission wavelengths. Subsequently those ^237^Np wavelengths have to be selected that are not suffering from spectral interference caused by the occurrence of other concomitant elements in the analyte solution. In the case of samples originating from experiments related to pyrochemical treatment of spent fuel, for example, the most sensitive ^237^Np emission line at *λ* = 382.92 nm cannot be employed at all because a serious spectral overlap from excess Nd, that is present is such samples, hampers the reliable determination of ^237^Np at this wavelength [2]. Trustworthy quantification of ^237^Np, however, can be carried out at the emission wavelengths *λ* = 410.84, 429.09 and 456.04 nm in such cases with results comparing well to SF-ICP-MS measurements carried out at m/z 237 [2]. If the investigated sample contains excess amounts of U, however, i.e. in the case of non-separated fuel solutions, both HR-ICP-OES and SF-ICP-MS analyses may suffer from spectral interferences requiring a separation of Np from the fuel matrix prior to analysis. Altogether the use of complementary instrumental techniques is highly recommended to ensure the accuracy of the obtained results, because no matrix-matched certified reference materials for the analysis of Np in spent fuel are currently available.

**Fig. 3:**
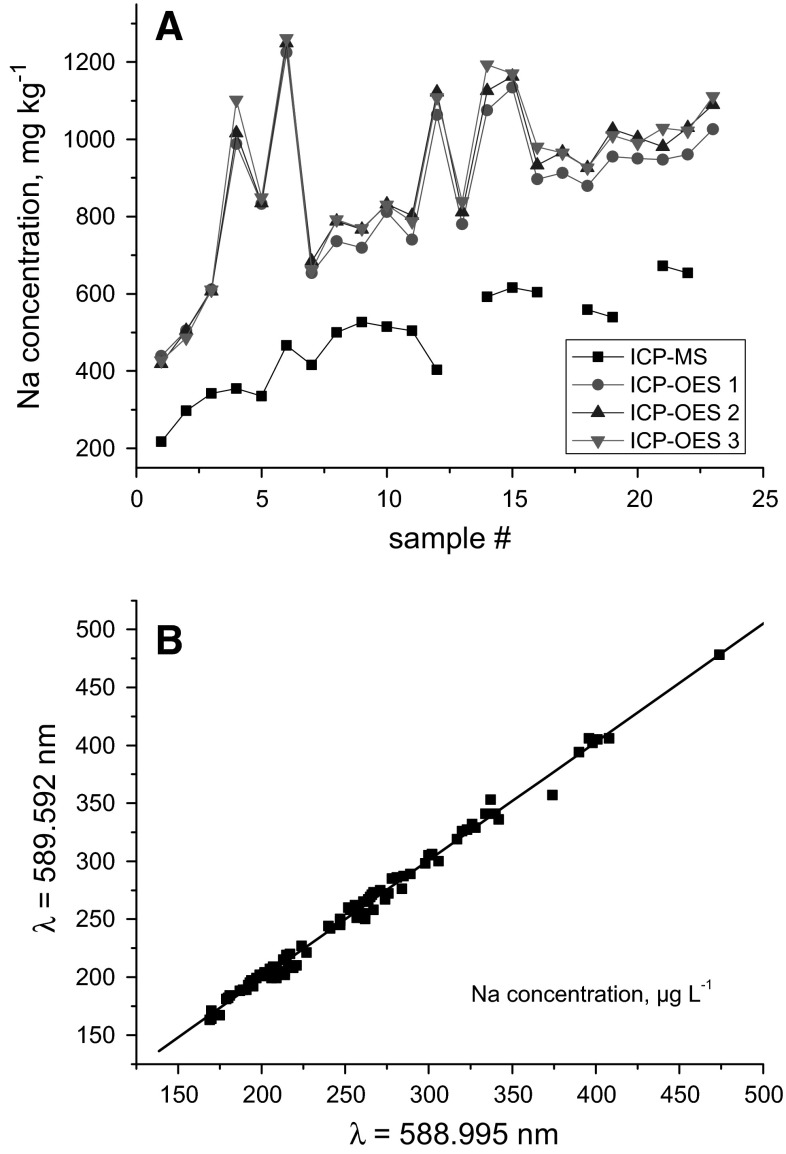
**a** Comparison of Na data in a suite of salt samples as determined by sector field ICP-MS and ICP-OES highlighting the high reproducibility of the ICP-OES results as well as the disagreement between both instrumental approaches. **b** Internally consistent Na concentration results obtained at two different emission wavelengths using HR-ICP-OES. All *data* from Ref. [17]. See text for details

During the development and optimisation of the electro-refining process of spent fuel in molten salts (which serve as solvent), it is also important to determine the concentration change of the major components of the employed alkali salts, i.e. Na, K, and Li. Using conventional ICP-MS with a quadrupole mass filter for this kind of analysis generates some serious troubles because the signal of the mono-isotopic Na at m/z 23 is interfered spectrally by the occurrence of the polyatomic species ^7^Li^16^O and ^6^Li^17^O (Li being present in 100-fold excess frequently), respectively, while the reliable determination of K suffers from spectral overlap caused by ^38^Ar^1^H at m/z 39 [17]. Sector field ICP-MS instruments, however, can overcome these problems by measuring Na in the medium resolution mode (*m*/Δ~ 4,000) and K in the high resolution mode (*m*/Δ*m* ~10,000). Besides, the use of the medium resolution mode also allows separating isobaric interferences of doubly charged ^12^C and ^14^N from ^6^Li and ^7^Li SF-ICP-MS signals, respectively [17]. This SF-ICP-MS approach for the analysis of the light elements Li, Na and K still remains troublesome because of the observed instrumental drift at the edge of the mass range of the magnet as well as the stability of mass calibration during long measurement sequences. Additionally, the accuracy of the SF-ICP-MS Na data suffers frequently from high Na blanks originating from handling samples in hot cell facilities using telemanipulators, resulting in high method detection limits. In general, these constraints and other difficulties limit the performance of SF-ICP-MS for the analysis of alkaline elements as showcased in Fig. 3a for Na and discussed in more detail elsewhere [17]. Results from the analysis of various certified water reference materials suggest that the Na data shown in Fig. 3a obtained via ICP-OES are accurate, while SF-ICP-MS results are less reliable (see below).

The robustness and sensitivity of HR-ICP-OES analysis, in turn, offer highly reproducible results and low detection limits (down to sub-µg kg^−1^ levels) for Na, K, and Li [17]. Concerning quality control, the use of certified water reference materials is justified for such analysis because the actual salt samples are diluted up to ~400,000-times for HR-ICP-OES analysis [17]. As such, the concentrations of the alkali elements are at similar levels in both the employed reference materials and the analyte solutions. The experimental concentrations established for Na, K, and Li in 3 different water reference materials agreed well with the certified values underpinning the accuracy of the applied HR-ICP-OES procedures [17].

For additional quality assurance, two emission wavelengths of the same element (e.g. Na) can be used to check for internally consistent results (Fig. 3b). The validity of the graphically sound results can be further proven mathematically by the confidence levels of the regression parameters being 1.019 ± 0.020 (slope) and −5.116 ± 5.299 (intercept) (*p* = 0.033). This data confirms that these parameters are not significantly different from 1 and 0. If similar results are obtained for the same element at two different wavelengths at least, this fact indicates the absence of spectral interferences underpinning the quality of the obtained HR-ICP-OES data [17].

Taken together, SF-ICP-MS measurements of the alkaline elements Na, K, and li were less stable than HR-ICP-OES analysis, revealing instrumental drifts that could only be partly compensated for. The HR-ICP-OES results, however, were highly reproducible and validated through the beneficial agreement between experimental and certified concentrations of Na, K, and Li in several certified reference materials [17]. The applied HR-ICP-OES procedures proved to be reliable, robust, more straightforward and less laborious than the SF-ICP-MS approach.

## Spent fuel analysis

Complementary analysis becomes especially important when dealing with spent fuel. Because of security and safety issues as well as expenses for the transport, spent fuel is normally not shipped to another laboratory for comparative analysis. Therefore, it is highly desirable to develop diverse analytical methods in-house that can be employed to cross-validate each other. Ideally, these analytical procedures can avoid laborious separation procedures, thereby also limiting the radiation dose to the analyst during sample preparation and speeding up the overall quantification step.

**Fig. 4:**
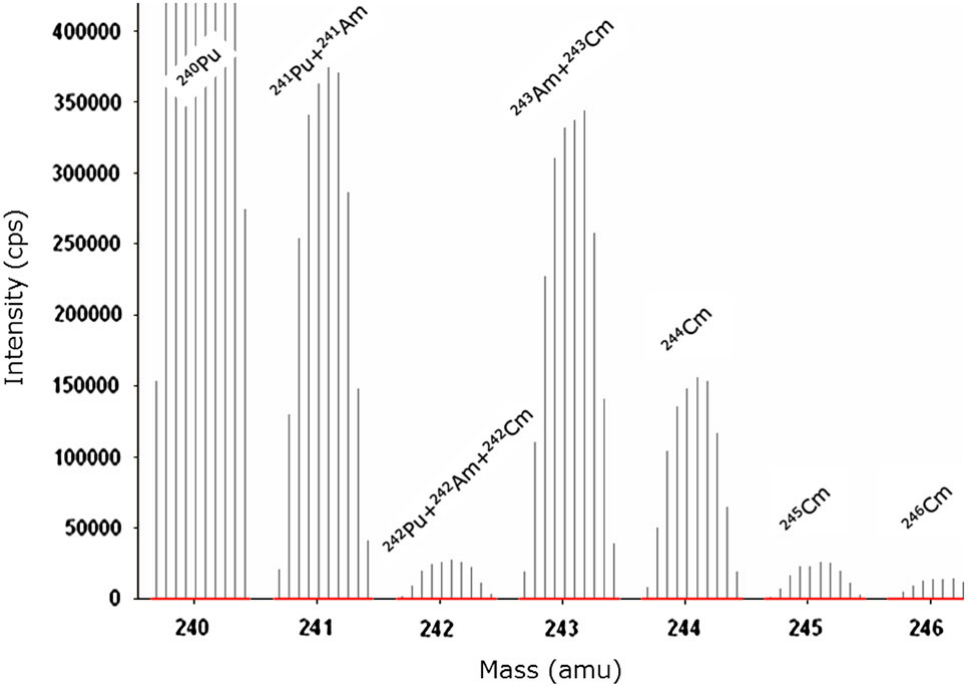
SF-ICP-MS spectrum from m/z 240 to 246 of a spent fuel sample revealing isobaric overlaps that hamper the reliable determination of Am [3]. See text for details

In that context, the direct analysis of Am in spent fuel using SF-ICP-MS and HR-ICP-OES may serve as a specific example. Besides ^241^Am, spent fuel always contains ^241^Pu hampering the reliable quantification of Am using ICP-MS (Fig. 4). As this isobaric overlap of the two isotopes cannot be resolved instrumentally, even with SF-ICP-MS instruments, additional information is required to identify the contribution of each isotope to the signal obtained at m/z 241 in the mass spectrum [3]. Knowledge of the initial nuclear fuel composition as well as the irradiation history provides essential input data to the ORIGEN-2 programme code that allows the calculation of the fuel composition after irradiation [28]. With this information at hand, spectral interferences (also Am, Cm, and Pu isotopes at m/z 242 and m/z 243) can be “resolved”, resulting in valuable isotopic and elemental Am information [3]. Even though this approach adds some uncertainty to the final result, a conservative estimate would state a corresponding uncertainty on the total Am concentration of about 5 % [3]. As such, SF-ICP-MS has the potential to provide accurate Am concentration and isotopic data without chemically separating off the element from the investigated spent fuel solution.

In the absence of an Am standard solution, however, calibration of the ICP-MS response is not trivial at all either. The closest element in the mass spectrum, for which a commercial standard solution can be purchased easily, is U. Calibrating the ICP-MS instrument for Am with this natural U standard solution, assumes that both elements behave very similarly during measurements though. While this assumption already gives reasonable results, the accuracy of the calibration strategy can be improved further by also taking into account the mass bias and oxide formation rate of Th and U, as described in detail elsewhere [3]. Briefly, this calibration methodology has been tested and validated through the analysis of various spent fuels [3]. The legitimacy of this experimental approach has been confirmed by the fact that the mean Am concentration for each spent fuel assessed via ICP-OES and ICP-MS differed at most by 4 % from each other [3].

Regarding ICP-OES, the above calibration strategy is not feasible at all because an Am standard solution is required for this instrumental approach. To this end, ^241^Am can be separated from a concentrated ^241^Pu solution using liquid extraction followed by extraction chromatography [4]. While the isotopic purity of the ^241^Am fraction can be checked via SF-ICP-MS as well as α- and γ-spectrometry, the latter allows the determination of its actual concentration [4]. With this well-characterised ^241^Am standard solution at hand, selective and sensitive ICP-OES Am emission lines were identified for spent fuel analysis [3]. Low detection limits (i.e. 0.07 µg kg^−1^ at *λ* = 283.226 nm) helped to increase dilution factors of actual spent fuel solutions, thereby keeping the radiation dose to the operator of the ICP-OES instrument as low as possible [3, 4]. Because ^241^Am and ^241^Pu emit light at different wavelengths, the isobaric spectral interference observed in the mass spectrum of ICP-MS, is not present in ICP-OES analysis. It is worth noting, however, that due to the complexity of the Am emission spectra and the much smaller isotopic shift of ^241^Am and ^243^Am compared to that of U isotopes, peak deconvolution strategies would be necessary to extract Am isotopic information from the HR-ICP-OES spectra. In other words, using HR-ICP-OES for Am analysis, the obtained emission signal reflects the sum of all Am isotopes, i.e. its concentration. Therefore, a direct ICP-OES determination of the Am concentration in spent fuel solutions is possible that can be used to cross-validate the results obtained via ICP-MS.

**Fig. 5:**
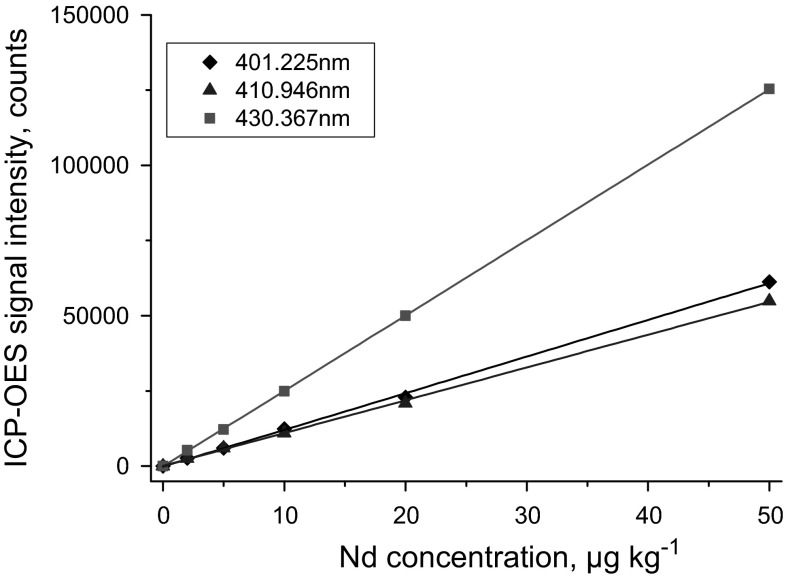
Representative ICP-OES calibration curves for neodymium (Nd) at the 3 emission wavelengths *λ* = 401.225, 410.946, and 430.367 nm highlighting the high sensitivity and performance of the upgraded instrumental set-up

Another interesting example comprises the determination of Nd that is commonly part of the radiochemical characterization of spent fuel because its isotopes serve as valuable burn-up indicators [29]. For Nd concentration analysis, calibration of HR-ICP-OES (Fig. 5) and subsequent quantification of Nd in spent fuel is straightforward [5]. Sensitive (sub-µg kg^−1^ detection limits) emission wavelengths, free from any spectral interference, have been identified successfully at *λ* = 401.225, 410.946, and 430.367 nm, for example [5]. As suitable reference materials are not available for the determination of Nd in spent fuel, the reliable assessment of the accuracy of the results is not trivial. In this context, the application of two different calibration strategies, namely external calibration and standard addition, may serve to help support the quality of the ICP-OES results. In fact, the comparison of the results obtained at the four most sensitive emission wavelengths revealed that both external calibration and standard addition gave very similar results (Table 3). As such, this agreement of the results between the two calibration approaches excluded the absence of potential matrix effects and spectral overlaps of concomitant elements in the analyte solution. However, an autonomous judgement of the quality of the ICP-OES data by another independent analytical approach, e.g. ICP-MS, is needed for quality assurance.

**Table 3:** Comparison of Nd concentrations (average ± SD, [µg kg^−1^]) in a solution of a dissolved spent fuel obtained via HR-ICP-OES analysis employing external calibration versus standard addition [5]

Wavelength (nm)	401.225	406.109	410.946	430.358
External calibration	32.4 ± 0.7	32.2 ± 0.9	32.0 ± 1.3	32.6 ± 1.5
Standard addition	31.9 ± 0.3	31.7 ± 1.3	32.0 ± 0.8	32.0 ± 1.2

The direct determination of the Nd concentration in spent fuel via ICP-MS is complicated by the fact that its Nd isotopic composition varies distinctly from the natural one. In addition, the ICP-MS signals of some Nd isotopes are overlapped by isobaric interferences (^142^Nd by ^142^Ce, ^148^Nd by ^148^Sm, and ^150^Nd by ^150^Sm) that cannot be resolved spectroscopically, even if SF-ICP-MS is employed. Additionally, the normally small isobaric contribution of ^144^Ce to the signal of ^144^Nd also needs to be known from γ-spectrometry.

Knowing the actual Nd isotopic composition (from separate measurements, for example), the ICP-MS signal of the four Nd isotopes at m/z 143, 144, 145, and 146 provides a solid basis for the reliable determination of the Nd concentration in spent fuel. Comparison of the ICP-MS data to the results obtained by ICP-OES again serves as a valuable tool for cross-validating an analytical approach that requires careful attention to guarantee high quality analytical results [5].

## Conclusions

This report highlighted some fundamental challenges related to the elemental and isotopic analysis of actinides and fission products in a variety of nuclear samples coming from different parts of the nuclear fuel cycle including nuclear forensics. The use of HR-ICP-OES and SF-ICP-MS provides accurate and precise analytical data relevant for all kind of issues related to nuclear security and safety.

Both instrumental approaches have an excellent potential for this endeavour, but their individual application may be limited by several distinct constraints in particular cases. Frequently, HR-ICP-OES and SF-ICP-MS complement each other in such difficult-to-analyse instances. While the brilliant performance of SF-ICP-MS for elemental and isotopic analysis is generally well accepted, HR-ICP-OES also serves as a powerful, yet underestimated, tool for such kind of analysis.

As it is much easier to analyse radioactive samples in-house than to ship them to another external laboratory, at least two independent analysis methods need to be available internally. In addition, it is essential to use certified reference materials for quality assurance, whenever this is possible. In the absence of such reference materials, the application of two independent analytical methods and different calibration strategies may be applied to support the quality of the acquired analytical data. Once accurate and precise analytical data are available, issues related to nuclear security and safety can be addressed accordingly.
